# Biological Activities and Phytochemical Screening of *Thuja occidentalis* Extracts with In Silico Approaches

**DOI:** 10.3390/ijms26030939

**Published:** 2025-01-23

**Authors:** Kareem Younes, Amr Abouzied, Saad Alqarni, Akram Elkashlan, Weiam Hussein, Rawabi Alhathal, Rahaf Albsher, Sarah Alshammari, Bader Huwaimel

**Affiliations:** 1 Department of Pharmaceutical Chemistry, College of Pharmacy, University of Hail, Hail 81442, Saudi Arabia; as.ibrahim@uoh.edu.sa (A.A.); s.alqarni@uoh.edu.sa (S.A.); w.hussein@liveuohedu.onmicrosoft.com (W.H.); b.huwaimel@uoh.edu.sa (B.H.); 2 Department of Biochemistry, Faculty of Pharmacy, University of Sadat City, Sadat City 32897, Egypt; akram.elkaslan@fop.usc.edu.eg; 3 College of Pharmacy, University of Hail, Hail 55476, Saudi Arabia; s201800546@uoh.edu.sa (R.A.); s201800603@uoh.edu.sa (R.A.); s201804619@uoh.edu.sa (S.A.); 4 Medical and Diagnostic Research Centre, University of Hail, Hail 55476, Saudi Arabia

**Keywords:** *Thuja occidentalis*, extracts, phytochemical composition, GS-MS analysis, biological activities, antiviral, anticancer, molecular docking

## Abstract

The evergreen coniferous tree *Thuja occidentalis* is a member of the Cupressaceae family. This study included biological, cytotoxic, and in silico docking analyses in addition to a phytochemical composition analysis of the plant leaves and stem ethanolic extracts. The extracts’ in vitro cytotoxicity efficacy against various cancer cell lines was examined. Additionally, certain phytochemical compounds were identified by gas chromatographic analysis and subsequently assessed in silico against anticancer molecular targets. Also, their antiviral effect was assessed. Good cytotoxic activity was demonstrated by plant extracts against the lung and colorectal cancer cell lines. With half-maximal inhibitory concentration values of 18.45 μg/mL for the leaf extract and 33.61 μg/mL for the stem extract, apoptosis and S-phase arrest was observed in the lung cancer cell line. In addition, the leaf extract demonstrated effective antiviral activity, with suppression rates of 17.7 and 16.2% for the herpes simplex and influenza viruses, respectively. Gas chromatographic analysis revealed the presence of relevant bioactive components such as Podocarp-7-en-3β-ol, 13β-methyl-13-vinyl, Megastigmatrienone, and Cedrol, which were tested in silico against anticancer molecular targets. Our findings suggest that plant ethanolic extracts may have potential therapeutic uses as anticancer drugs against lung cancer in addition to their antiviral properties, which opens up further avenues for more investigation and applications.

## 1. Introduction

Complementary therapies are increasingly being used as a therapeutic approach. In this sense, the utilization of plants as remedies for a variety of illnesses is significant. Herbal medications are gaining popularity globally for several reasons, including their long-lasting therapeutic effects, safety, effectiveness, and low adverse effects [1].

Natural products continue to be the best sources of medications and drug leads because they are molecules that have evolved to be more like pharmaceuticals [2]. More than one hundred new products are undergoing clinical development, with a focus on anti-infective and anticancer drugs. Additionally, combinatorial chemistry approaches are being based on natural product scaffolds to create screening libraries that closely resemble drug-like compounds [3].

In the quest to develop drugs that help alleviate human suffering, medicinal plants present a fantastic opportunity to discover novel pharmacophores. Nowadays, historically used medicinal plants have garnered a lot of attention and are a major target for drug research. Additionally, they make up a sizable amount of the global pharmaceutical sector [4]. Indolin-2-one derivatives are examples of discovered bioactive natural compounds that possess promising anti-breast cancer activity [5].

One of the productive coniferous trees, *Thuja occidentalis* (*T. occidentalis*), is used to treat a variety of ailments with its oil and leaves. It is native to regions of Europe and southern Britain [6]. West Asia is home to a variety of plant species, including those found in different regions of the Kingdom of Saudi Arabia [7]. *T. occidentalis* has been demonstrated to exhibit anti-inflammatory [8], antitumoral [9,10,11], antioxidant [12], antibacterial and antifungal [13,14], antidiabetic [15], hypolipidemic and atheroprotective [16], gastroprotective [17], and antiviral and immune-stimulatory [18] activities. The plant’s immune-stimulating and antiviral properties have been shown [19,20]. Using both in vitro and in vivo models, Torres et al. investigated the effects of thujone on glioblastoma. Researchers have discovered that thujone has antiproliferative, proapoptotic, and antiangiogenic properties in vitro that may lower cell viability [9]. Additionally, thujone showed promise in stopping the spread of melanoma in living things [21].

Furthermore, polysaccharides extracted from the plant increased the activity of complement-mediated cytotoxicity (ACC) and cell-mediated antibody-dependent cytotoxicity (ADCC), as well as TIMP, IL-2, anticancer factors, and natural killer (NK) cells [10]. This tree’s leaves have also been utilized for their antibacterial and antioxidant qualities [14,22,23]. It has been demonstrated that *T. occidentalis* possesses antibacterial properties against a wide range of Gram-positive and Gram-negative bacteria [24]. Moreover, *T. occidentalis*’ antifungal properties have been observed to target various fungi such as Aspergillus [25].

In the current work, our aim is to evaluate the biological activities of *T. occidentalis*’ ethanolic extracts of leaves (TOL) and stem (TOS), such as their antiviral effects and anticancer capabilities against various cancer cell lines together with the exploration of their apoptotic effect. Also, this work describes phytochemicals identified in TOL through the gas chromatography–mass spectrometry (GC-MS) analysis technique. To investigate the potential of bioactive phytochemicals to suppress the proliferation of cancer cell lines, additional in silico docking studies were conducted against anticancer molecular targets. Our research opens up new possibilities for studying its industrial applications and may offer a natural treatment for patients with lung cancer.

## 2. Results

### 2.1. GC-MS Analysis

**Figure 1:**
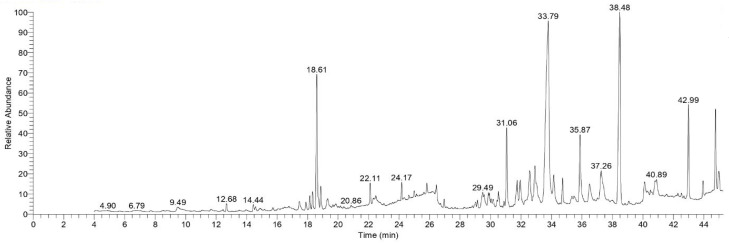
GC-MS chromatogram of TOL extract.

Several peaks were observed in TOL extract GC-MS chromatogram. These peaks may relate to bioactive substances. By contrasting these peaks’ mass spectral fragmentation patterns with those of the established standards, these peaks were further identified. As shown in Figure 1 and Table 1.

### 2.2. Biological Activities

#### 2.2.1. Antiviral Assay

Both influenza A (H1N1) and herpes simplex (HSV-2) viruses were efficiently suppressed by TOL extract at a concentration of 100 μg/mL, exhibiting respective viral inhibition rates of 17.7% and 16.2%. Both rates are comparable to those of standard ribavirin (24.5 and 22.4% for the HSV-2 and H1N1 viruses, respectively).

Table 2 displays the IC_50_ values for the HSV-2 and H1N1 viruses. For the HSV-2 virus, these values were found to be 305 μg/mL and 912 μg/mL, respectively, compared to 282 μg/mL and 867 μg/mL of standard ribavirin, respectively. Table 2 illustrates the evaluation of the TOS extract’s antiviral activity against the HSV-2 and H1N1 viruses.

#### 2.2.2. Cytotoxicity Studies

**Figure 2:**
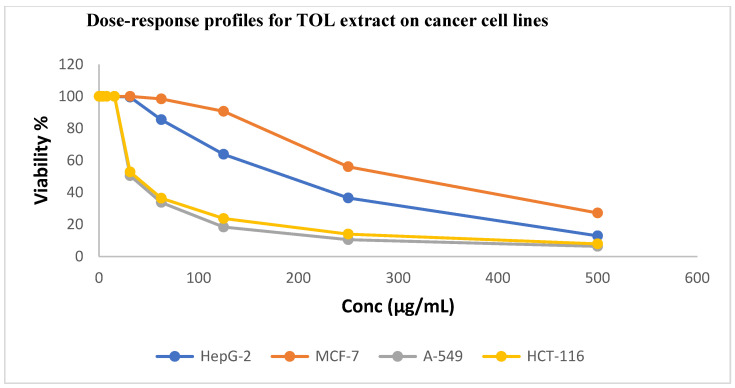
Dose–response profiles showing cytotoxic activity of TOL extract against investigated cancer cell lines.

**Figure 3:**
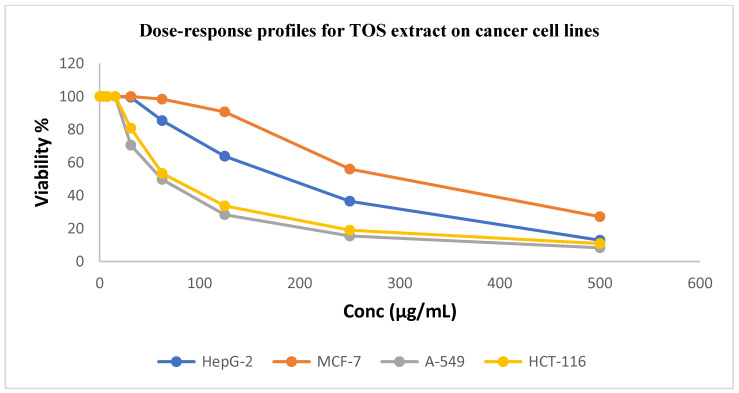
Dose–response profiles showing cytotoxic activity of TOS extract against investigated cancer cell lines.

As illustrated in Figure 2 and Figure 3, TOL and TOS extracts were evaluated against the HepG2, A549, HCT116, and MCF7 cancer cell lines. The A549 and HCT116 cell lines were found to be significantly susceptible to the powerful cytotoxic activity of the leaf extract, with IC_50_ values of 18.45 ± 1.2 and 24.90 ± 1.7 μg/mL, respectively. With IC_50_ values of 1.44 ± 0.3 μg/mL and 1.16 ± 0.2 μg/mL for the A549 and HCT116 cell lines, respectively, this activity is comparable to that of standard doxorubicine.

Table 3 indicates that TOS extract has moderate cytotoxic activity against the A549 and HCT116 cancer cell lines, with IC_50_ values of 33.61 ± 2.0 and 45.30 ± 2.4 μg/mL, respectively, in comparison to standard doxorubicin (1.44 ± 0.3 and 1.16 ± 0.2 μg/mL, respectively). Each of the two extracts showed a negligible cytotoxic effect on the cancer cell lines HepG2 and MCF7.

The A549 and HCT116 cancer cell lines were significantly more affected by the leaf extract than were MRC5 cells, which are normal cells. The A549 cancer cell line exhibited the highest level of selectivity, as Table 4 demonstrates, with leaf and stem extracts being almost three times more selective.

##### In A549 Cells, TOL Extract Produced S-Phase Cell Cycle Arrest

**Figure 4:**
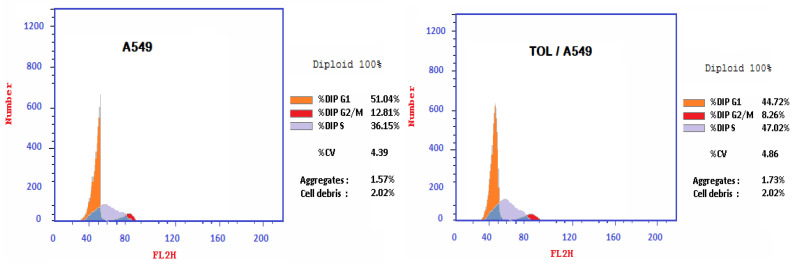
Effect of TOL extract on A549 cancer cell line-cycle after 72 h of treatment at IC_50_ concentration.

The percentage of A549 cancer cells in the S-phase rose when the TOL extract was applied, as seen in Figure 4 (47.2% compared to 36.15% in untreated cells). This increase in the percentage of cells in the S-phase relative to the control indicates that the TOL extract induces S-phase cell cycle arrest in the lung cancer cell line.

##### TOL Extract Induced Caspase-Dependent Apoptosis in A549 Cancer Cell Line, Which Was Mediated by p53

**Figure 5:**
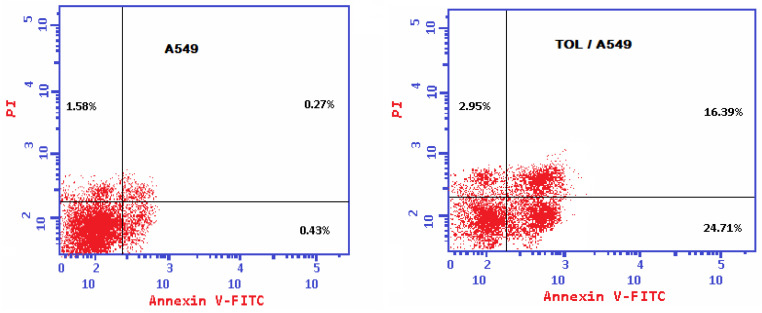
Annexin V/PI apoptosis assay images demonstrating the apoptosis-inducing effects of TOL extract on A549 cancer cell line after 72 h of treatment at IC_50_ concentration.

The TOL extract caused early (24.71% against 0.43% in the untreated cells) and late (16.39% versus 0.27% in the untreated cells) apoptosis. Thus, it is fair to conclude that the TOL extract primarily killed cancer cells by generating apoptosis as shown in Figure 5.

The Western blot analysis results demonstrated that the administration of the TOL extract to the A549 cancer cell line produced significant amounts of cleaved caspase-3 protein production. This is consistent with the Annexin V/PI assay results displayed in Table 5 and explains why the treated A549 cancer cell line experienced apoptosis.

### 2.3. Molecular Docking

After evaluating the binding affinities of several screened phytochemical constituents with 4-{[4-(1-benzothiophen-4-yloxy)-3-chlorophenyl]amino}-N-(2-hydroxyethyl)-8,9-dihydro-7H-pyrimido[4,5-b]azepine-6-carboxamide (W19) [49] (ΔG of −10.5 kcal/mol), β-Sitosterol showed the highest binding affinity, outperforming the reference standard (W19) with a ΔG of -10.6 kcal/mol.

**Figure 6:**
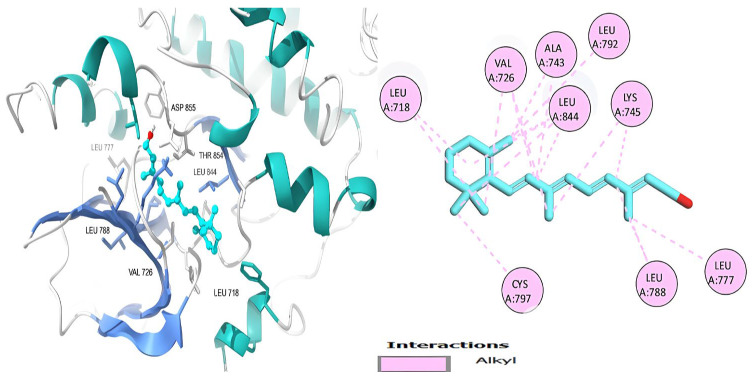
Retinol’s 3D and 2D molecular interactions with EGFR TK residues.

**Figure 7:**
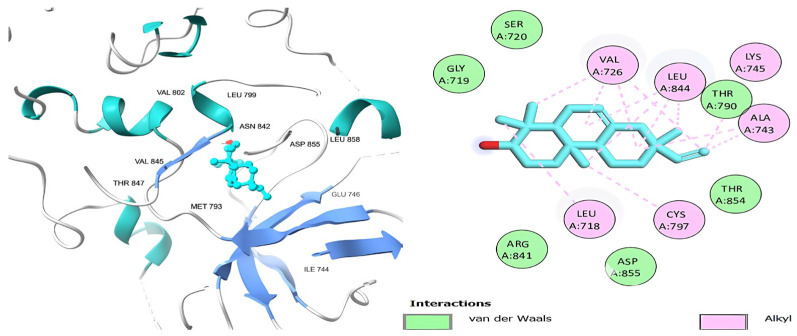
Podocarp-7-en-3β-ol, 13β-methyl-13-vinyl’s 3D and 2D molecular interactions with EGFR TK domain residues.

**Figure 8:**
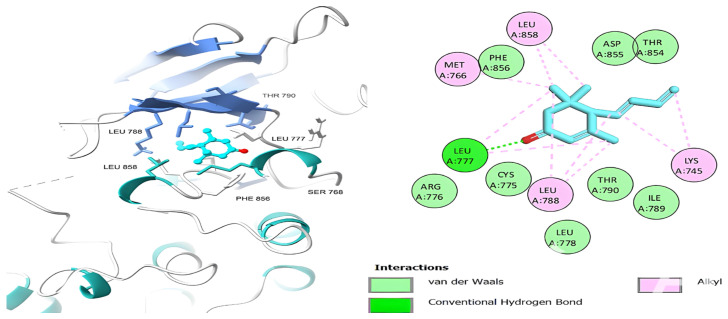
Megastigmatrienone’s 3D and 2D molecular interactions with EGFR TK domain residues.

**Figure 9:**
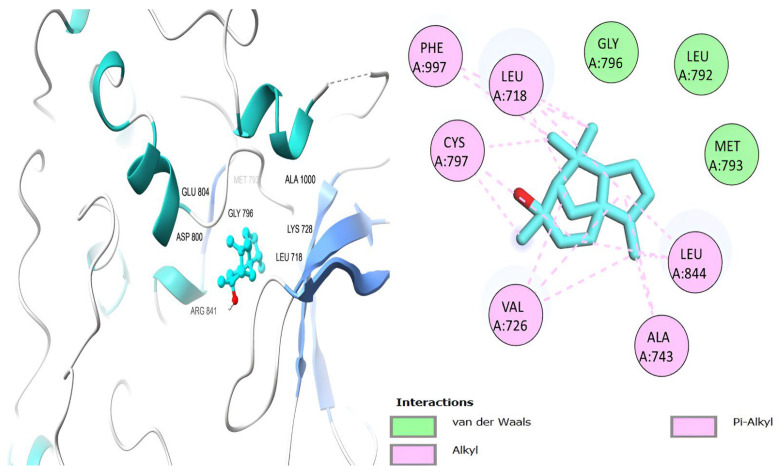
Cedrol’s Interactions with EGFR TK domain residues in two and three dimensions.

**Figure 10:**
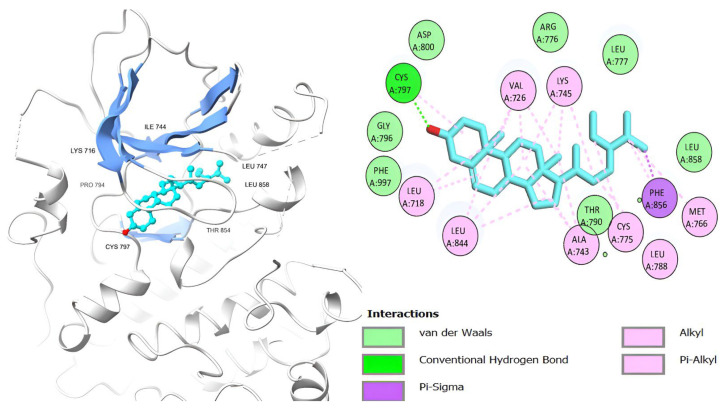
β-Sitosterol’s 3D and 2D molecular interactions with EGFR TK domain residues.

**Figure 11:**
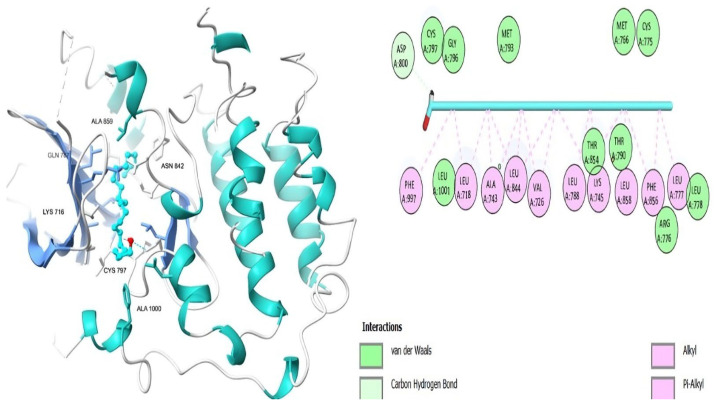
Docosanol’s 3D and 2D molecular interactions EGFR TK domain.

Table 6 shows that the amino acids LYS 745, MET 793, GLY 796, ASN 842, ASP 855, LEU 718, PHE 723, VAL 726, ALA 743, THR 790, LEU 844, and THR 854 actively participate in the interactions as a result of the in silico protein-screened phytochemical component interaction. The screened phytochemical elements displayed binding energies ranging from ΔG −6.2 to −10.6 kcal/mol, as seen in Table 6 and Figure 6, Figure 7, Figure 8, Figure 9, Figure 10 and Figure 11. This suggests a potential for interactions with the active sites of Epidermal Growth Factor Receptor Tyrosine Kinase Domain (EGFR TK).

## 3. Discussion

From the GC-MS analysis of the TOL extract, many bioactive molecules were identified. Some of them were responsible for the bioactivity of plant extracts such as: Retinol, Podocarp-7-en-3β-ol, 13β-methyl-13-vinyl, Megastigmatrienone, Cedrol, β-Sitosterol, and Docosanol. Retinol is a biologically active substance that possess antioxidant properties. It stimulates the activity of fibroblasts to produce collagen and, in addition, it improves skin elasticity and promotes angiogenesis [26]. Podocarp-7-en-3β-ol, 13β-methyl-13-vinyl is a chemical substance that increases the inhibition of cancer cell growth [27]. Megastigmatrienone is a main component of terpenes that play an important role in decreasing cancer cell growth [28]. Cedrol is a natural sesquiterpene substance that has antibacterial [29] and antitumor activities, especially against colorectal cancer [30]. β-Sitosterol is a chemical substance that interferes with cell proliferation, survival, and cell cycle apoptosis that induce an anticancer effect [31]. Docosanol is a saturated alcohol that exerts inhibitory effect on virus replication [32].

The effectiveness of *T. occidentalis* extracts as antiviral agents against the HSV-2 and H1N1 viruses was studied. Both H1N1 and HSV-2 are highly pathogenic viruses and one of them (H1N1) can cause seasonal epidemics. The results indicate that TOS, at 100 μg/mL, had a mild action against both viruses, with low viral suppression rates which were weakly comparable to those of standard ribavirin, while it had no antiviral action at 50 μg/mL.

The condition known as cancer is caused by unchecked cell division that invades nearby tissue. Changes in DNA are most likely the cause. “Cytotoxic drugs” are anticancer medications that either directly alter DNA or interfere with cell division to damage protein synthesis. We investigated the anticancer effect of plant extracts on a variety of cancer cell lines in vitro. In vitro and in vivo models of glioblastoma have been reported to be impacted by plant thujone components in earlier research [9]. Animal models of cytokine levels and cell-mediated immune responses in metastatic tumors have been used to study the plant and its polysaccharides [10]. The plant extracts’ anti-metastatic properties have been studied in mice that had had melanoma induced [11]. The high selectivity of both extracts implies that they cause less harm to healthy cells. With these positive outcomes against the A549 cancer cell line, we looked into the mechanism of action of *T. occidentalis* leaf extract on cancer cell lines.

To understand how the plant extract works as an anticancer agent, the TOL extract was tested for its ability to induce apoptosis and a state of cell arrest by measuring checkpoint levels. The IC_50_ of the plant extract was investigated in relation to lung cancer cell lines. The findings of Western blot analysis suggest that the A-549 cancer cell line experiences apoptosis in response to the TOL extract. During an examination of the A-549 cell cycle, S-phase arrest and the initiation of apoptosis were found.

The TOL extract induces apoptosis, as evidenced by the expression levels of several apoptosis-related proteins, such as caspase-3 and p53. The caspase protein family is primarily responsible for controlling apoptosis. One way to think of caspases, such caspase-3, is as often-activated death proteases. The caspase–cascade system illustrates how numerous molecules control caspase activation and function, including calpain, apoptosis protein inhibitor, Bcl-2 family proteins, and Ca^2+^ [50]. P53 is one of the proteins that has anti-proliferative qualities because it prevents tumors from growing [51].

Bcl-2, an anti-apoptotic protein, can inhibit Bax’s activities since it is believed to be a p53 target and is responsible for activating caspase during apoptosis [52,53]. Therefore, analyzing these proteins might shed light on how the leaf extract initiates the apoptotic process. The results of the Western blot analysis showed that, in the A-549 cancer cell line, the leaf extract induced the expression of the Bax protein and decreased the expression of Bcl-2. As Table 5 shows, this implies that the leaf extract has pro-apoptotic effects and this is in line with the results of previous experiments pertaining to apoptosis.

The EGFR TK protein was selected for the molecular docking study because it has the ability to be inhibited, which limits the growth pathway and offers a possible anticancer medication [54]. A lower binding energy resulting from the compound’s interaction with the intended protein indicates a higher binding efficiency. W19 acted as an EGFR TK inhibitor in this study.

## 4. Materials and Methods

### 4.1. Chemicals and Solvents

All solvents and chemicals used were of the highest grade and were obtained from Sigma-Aldrich (St. Louis, MO, USA). Ethanol 70%, dimethylsulfoxide (DMSO) 99.7%, Formaldehyde (>36%), crystal violet (P.N. C6158), Dulbecco’s modified Eagle medium (DMEM) (P.N., D6046), 4′,6-diamidino-2-phenylindole (DAPI) (P.N. D8417), and Phosphate-buffered saline (PBS) (P.N. 806552) were used. A liquid sterile-filtered medium; Roswell Park Memorial Institute Medium (RPMI-1640), was used. All cell culture reagents and the MTT kit were obtained from Biowest-The Serum Specialist, Nuaillé, France.

### 4.2. Collection and Extraction of Plant Material

In the Saudi Arabian province of Hail, *T. occidentalis* was collected in spring and identified by Dr. Naila Hassan Alkefai, University of Hafr Al-Batin, and a voucher specimen (No. UOHCOP022) has been deposited at the College of Pharmacy, University of Hail. The leaves and stem were separated and each was ground and converted into a powder. The leaves and stem powder (25 g of each) were separately macerated in 200 mL 70% ethanol for 3 days and the extract was later filtered and concentrated via a rotary evaporator. TOL and TOS extracts were collected.

### 4.3. GC-MS Analysis

Upon using the GC-MS technique, more details were obtained on the investigated ethanolic extract. The ideal chromatographic condition was determined by utilizing Thermo MS-DSQ II and Thermo GC-TRACE extreme version 5.0 (Thermo Scientific, Austin, TX, USA) on a non-polar capillary column with an ID of a 0.25 mm thickness and a length of 30 m.

The TOL extract was chemically evaluated using a Trace GC-TSQ mass spectrometer. The column oven’s temperature was first set at 50 °C, increased by 5 °C each minute to 250 °C, and then maintained for two minutes. At a rate of 30 °C per minute, the temperature was then raised to 300 °C and held there for two minutes while using a 10 μL injection volume. The MS transfer line was maintained at 260 °C and the injection unit at 270 °C. Helium was used as a carrier gas for the duration of the investigation, with a constant flow rate of 1 mL/min. The mass spectra were interpreted using NIST, version 1.0 software library in the *m*/*z* range of 0–700.

For preparing the extract for GC-MS analysis, a sample of dried leaf powder was extracted by ethanol over the course of 72 h. The same solvent was used for many extractions until a clear, colorless solvent was produced. The obtained extract was dried by evaporation and kept for GC-MS analysis at 4 °C in an airtight container.

### 4.4. Biological Assays

#### 4.4.1. Antiviral Activity Assay

##### Viruses

Viral strains were obtained from virus stock present in the regional center for Mycology and Biotechnology, Al-Azhar University, Cairo, Egypt. Based on their importance in human infection, we selected the H1N1 (VR-1736) and HSV-2 (VR-540) viruses. Each viral strain was incubated for twenty-four hours at 37 °C.

##### Half-Maximal Cytotoxic Concentration (CC50) Determination

The examined TOL and TOS extracts were diluted to working solutions with DMEM after stock solutions were prepared in 10% DMSO in ddH_2_O to determine the half-maximum cytotoxic concentration (CC_50_). The crystal violet test [55] was used to measure the material’s cytotoxic activity in Madin–Darby canine kidney (MDCK) cells. The cells were cultivated in 96-well plates with 100 µL/well and a density of 3 × 10^5^ cells/mL for 24 h at 37 °C and 5% CO_2_. The investigated extracts were given to the cells in triplicate, at varying concentrations (0, 10, 100, 200, 500, and 1000 μg/mL), after a day [56].

After 72 h, the cell monolayers were fixed for 1 h at room temperature (RT) with 10% formaldehyde, and the supernatant was separated. Following appropriate drying, the fixed monolayers were colored for 20 min at room temperature using 50 mL of 0.1% crystal violet. Following washing and the overnight drying of the monolayers, 200 mL of methanol was used to dissolve the crystal violet dye present in each well, and the mixture was let to remain at room temperature for 20 min. Using a multi-well plate reader (ImmunoSpot^®^, Cleveland, OH, USA), the absorbance of the crystal violet solutions was determined at a maximum wavelength of 570 nm. GraphPad Prism software version 5.01 (DOTMATICS, Woburn, MA, USA) was used to perform a nonlinear regression analysis and determine CC_50_ value.

##### Half-Maximal Inhibitory Concentration (IC50) Determination

The IC_50_ test was carried out as previously mentioned [57]. Following their separation into 96-well tissue culture plates, 3 × 10^5^ MDCK cells were incubated for a whole night at 37 °C with 5% CO_2_. Following a single PBS wash, the cell monolayers were exposed to vesicular stomatitis virus adsorption for an hour at room temperature.

An extra 50 μL of DMEM with varying concentrations (0, 10, 100, 200, 500, and 1000 μg/mL) of the investigated TOL or TOS extracts was added on top of the cell monolayers. The cells were incubated for 72 h at 37 °C in a 5% CO_2_ incubator. Following this, they were fixed for 20 min with 100 μL of 4% paraformaldehyde. After that it was stained for 15 min at room temperature with 0.1% crystal violet in distilled water. After dissolving the crystal violet dye in 100 μL of 100% methanol in each well, the optical density of the color was measured at 570 nm using an Anthos plate reader. The amount of the substance required to reduce the virus-induced cytopathic effect (CPE) relative to virus control is known as the half-maximal inhibitory concentration (IC_50_).

#### 4.4.2. Cytotoxic Activity Assay

##### Cancer Cell Lines

The MCF7 cancer cell line (ATCC No. HTB-22^TM^-human breast cancer cell line), HepG2 cancer cell line (ATCC No. HB-8065^TM^ hepatocellular carcinoma cell line), HCT116 cancer cell line (ATCC No. CCL-247^TM^ Colon carcinoma cell line), and A549 cancer cell line (ATCC No. CCL-185^TM^-human lung carcinoma) were purchased from the American type culture collection (ATCC, Rockville, MD, Manassas, VA, USA). The cancer cell lines were grown in RPMI-1640 media supplemented with inactivated 10% FBS and gentamycin (50 µg/mL). The cells were incubated at a humid atmosphere at a temperature of 37 °C with 5% CO_2_ and were sub-cultured twice or thrice per week.

##### Cell Viability Assay

The assay for cell viability can indicate metabolic activity and healthy cell processes, which in turn can be used to detect cytotoxicity. The cancer cell lines were arranged at a density of 5 × 10^4^ cells/well on Corning^®^, (Corning Incorporated, Somerville, MA, USA) 96-well tissue culture plates in accordance with the 3-[4,5-dimethylthiazol-2-yl]-2,5 diphenyl tetrazolium bromide (MTT) assay technique for cell viability and proliferation. The plates underwent a full day of incubation. Then, twelve concentrations of the combined three-extract replicates were applied to each cell line. The control was a 96-well plate filled with 0.5% DMSO or medium. Following a 72 h incubation period, 10 μL of the MTT reagent was added to each well, and the MTT assay procedure was followed to determine the number of viable cells [58].

##### Apoptosis Analysis (Annexin V-FITC Assay) of A549 Cells

Using flow cytometry equipment and Annexin V-FITC kits (Thermo Fisher Scientific, Waltham, MA, USA) apoptosis in the A549 cells was examined. The Annexin V-FITC assay was used to further evaluate apoptotic cells. As instructed, the TOL extract was applied to grown A549 cells at its IC_50_ concentration (18.45 μg/mL). Following a 72 h treatment period, the A549 cells were collected, rinsed twice in PBS for 20 min each, and then rinsed with binding buffer.

Furthermore, 1 mL of FITC-Annexin V was added to 100 mL of kit binding buffer containing suspended cells. After that, it was incubated for 40 min at 4 °C. Following a wash, the cells were again suspended in 150 mL of binding buffer containing 1 mL of DAPI (1 μg/mL in PBS).

##### Cell Cycle Analysis Using Flow Cytometry

Cell cycle analysis was performed to investigate the cell cycle distribution of the A549 cancer cell line in order to assess the effect of the tested extract. To do that, DNA was stained with a fluorescent dye and its intensity was assessed as part of a cell cycle analysis technique. Since the dye stains DNA stoichiometrically, it is possible to identify aneuploidy populations and differentiate between cells in G0/G1, the S phase, and G2/M. The overall analysis remains the same even though different sample types might be dyed using different techniques [59].

##### Western Blot Analysis

Western blotting is an essential technique in cell and molecular biology. Using a Western blot, researchers can identify specific proteins from a complex mixture of proteins from cells. This is accomplished by the three-step procedure, which includes size separation, transfer to a solid substrate, and target protein tagging with the relevant primary and secondary antibodies for its visualization by fluorescent detection [60].

### 4.5. Molecular Docking

The Molecular Operating Environment (MOE) 2019.012 suite (Chemical Computing Group, ULC, Montreal, QC, Canada) [61] was utilized in docking experiments to compare the binding scores and modes of the screened metabolites to W19 [49], which served as the reference standard. This made it possible to propose that the substances work as protein inhibitors of the EGFR TK.

After being added to the MOE window, the screened phytochemical components experienced partial charge addition and energy minimization [62]. The produced compounds were saved as a Microsoft Access Database (MDB) file, which needed to be added to a database using W19 and placed into the docking step’s ligand icon.

The Protein Data Bank provided the target X-ray for the EGFR TK [63]. It was also readied for the docking approach by strictly following the previously outlined steps [64,65]. Notably, the downloaded protein was energy-minimized, error-corrected, and featured 3D hydrogens [66,67]. Using a conventional docking procedure, the screened metabolites were used to replace the ligand site. Following the previously mentioned adjustment of the default program requirements, the docking process was started [68].

The docking location was selected to be the co-crystallized ligand site. In short, the docking site was chosen using the fake atoms method. The placement and scoring systems that were employed were Triangle Matcher and London dG, respectively. For each docked molecule, the rigid receptor and GBVI/WSA dG scoring techniques were utilized to extract the top 10 poses out of 100 poses [69,70]. The optimal pose for more research was determined using the highest acceptable score, each ligand’s binding mode, and its root mean square deviation (RMSD) value.

It is important to keep in mind that, as part of the program validation phase for the used MOE program, the co-crystallized ligand-W19 [49] was re-docked to its binding pocket of the produced target [71,72]. The initial figure, a low RMSD value of 1.22 between the tested metabolites and the re-docked co-crystallized ligand (W19) indicated a valid performance. The MOE program’s outcomes were further illustrated using Discovery Studio 4.0 software.

### 4.6. Statistical Analysis

To obtain data that were statistically significant, each measurement was performed in triplicate. Graph Pad Prism statistical software version 7 (DOTMATICS, Woburn, MA, USA) was used to express the data as an average value (mean) ± standard deviation (SD) at *p* < 0.05.

## 5. Conclusions

This study investigated the anticancer and antiviral potential of *Thuja occidentalis* ethanolic extracts in detail. The stem and leaf extracts were separately studied. The leaf extract exerted much higher cytotoxic activity than the stem extract against the A-549 and HCT116 cancer cell lines with the highest activity being observed against the A549 cancer cell line. Further studies showed that leaf extract caused the A549 cancer cell line to experience cell death via the induction of apoptosis and S-phase cell cycle arrest with very little cytotoxic action against normal MRC5 cells. This may highlight its potential as an effective and safe anticancer agent.

Moreover, the leaf extract was found to possess higher antiviral activity than the stem extract, and this activity was even comparable to that of standard ribavirin. A bioassay guided GC-MS analysis was carried out and many bioactive molecules were identified. A further in silico docking study was performed that revealed promising interactions between some identified bioactive compounds and molecular protein targets which confirms their pharmacological relevance.

These results support the extract’s potential use as a natural alternative or adjuvant treatment for lung cancer and offer fresh insights into the molecular mechanisms behind the extract’s anticancer effect. In order to precisely separate the molecules causing the reported bioactivities of the plant extracts and try to turn them into a useful medication with additional in vivo testing using animal models, bioassay-guided isolation studies are anticipated to follow this work.

## Figures and Tables

**Table 1:** The major chemical composition of TOL.

GC-MS Analysis of TOL					
Plant Extracts	Compound Name	M.Wt (amu)	RT (min)	Area %	Biological Activity
TOL	Retinol	286	35.87	4.38	Antioxidant effect [26]
	Podocarp-7-en-3β-ol, 13β-methyl-13-vinyl	288	31.06	4.65	Anticancer effect [27]
	Megastigmatrienone	190	18.86	1.27	Antiproliferative effect [28]
	Cedrol	222	18.33	1.01	Antibacterial, antitumor effect [29,30]
	β-Sitosterol	414	44.76	4.28	Anticancer effect [31]
	Docosanol	326	42.99	5.39	Antiviral effect [32]
	Cedrene	204	14.44	0.46	Anticancer effect [33]
	3-tert-Butyl-4-hydroxyanisole	180	17.47	0.75	Antihyperlipidemia [34]
	Caryophyllene oxide	220	18.15	0.78	Anticancer and analgesic [35]
	Gleenol	222	19.32	0.70	Termiticidal, antihelminitic [36]
	Cedryl acetate	264	22.11	1.15	Anti-obesity [37]
	Tetradecanoic acid	228	22.48	0.64	Antibacterial and antivirulence [38]
	Neophytadiene	278	24.17	0.88	Antidepressant and sedative [39]
	n-Hexadecanoic acid	256	26.46	0.83	Antioxidant [40]
	5,8,11,14-Eicosatetraenoic acid, methyl ester	318	29.48	1.08	Antimicrobial and antiinflammatory [41]
	trans-13-Octadecenoic acid	282	29.58	0.49	Antioxidant [42]
	Androstan-3-one, 17-(Acetyloxy)-, (5à,17á)	332	30.18	0.37	Antimicrobial activity [43]
	1-Phenanthrenemethanol	286	31.75	1.73	Cytotoxic and antiinflammatory [44]
	Podocarp-7-en-3-one	286	31.95	1.46	Antifungal [45]
	Pimaric acid	302	32.92	2.48	Antibacterial [46]
	Lutein	568	40.8	0.95	Antioxidant, antiinflammatory [47]
	Campesterol	400	43.94	0.91	Anticancer, cholesterol lowering [48]

**Table 2:** IC_50_ values of *T. occidentalis* extracts’ antiviral activity.

Plant Extract	% Inhibition of Virus		
	HSV-2	H1N1	Standard Ribavirin
TOL			
Concentration (100 μg/mL)	17.70	16.20	24.50 (HSV-2)
			22.40 (H1N1)
Concentration (50 μg/mL)	8.10	6.70	12.10 (HSV-2)
			10.70 (H1N1)
IC_50_ * (μg/mL)	305 ± 3.6	912 ± 2.7	282 ± 1.9 (HSV-2)
			867 ± 1.7 (H1N1)
TOS			
Concentration (100 μg/mL)	3.8	2.1	24.50 (HSV-2)
			22.40 (H1N1)
Concentration (50 μg/mL)	0	0	12.10 (HSV-2)
			10.70 (H1N1)
IC_50_ * (μg/mL)	1530 ± 3.7	1790 ± 4.0	282 ± 1.9 (HSV-2)
			867 ± 1.7 (H1N1)

* IC_50_ values are reported as the mean (IC_50_ ± SD) of three experiments.

**Table 3:** IC_50_ values of *T. occidentalis* extracts against investigated cancer cell lines.

IC_50_ (μg/mL) ^a^					
		HepG2	A549	HCT116	MCF7
Extract	TOL	>100	18.45 ± 1.2	24.90 ± 1.7	>100
	TOS	>100	33.61 ± 2.0	45.30 ± 2.4	>100
Doxorubicin		1.17 ± 0.1	1.44 ± 0.3	1.16 ± 0.2	2.39 ± 0.3

^a^ IC_50_ values are reported as the mean (IC_50_ ± SD) of three experiments.

**Table 4:** Selectivity index of *T. occidentalis* extracts.

Selectivity Index (SI) ^a^					
		HepG2	A549	HCT116	MCF7
Extract	TOL	--------	3.70	2.74	--------
	TOS	--------	3.92	2.90	--------
MRC5 Leaves		68.32 ± 2.1			
IC_50_ ^b^ (μg/mL) Stem		131.78 ± 3.0			

^a^ SI = (IC_50_ of MRC5)/(IC_50_ of cancer cell line), ^b^ IC_50_ values are reported as the mean (IC_50_ ± SD) of three experiments.

**Table 5:** Effect of TOL extract on apoptotic proteins of A549 cancer cell line.

Samples	Protein Expression * (μg/mL)			
	Bax	BCl2	Caspase-3	P-53
Control (A549 cancer cell line—untreated)	3.20 ± 0.4	7.53 ± 0.4	69.31 ± 3.1	3.81 ± 0.2
TOL	14.20 ± 0.4	3.95 ± 0.3	141.85 ± 4.0	11.90 ± 1.7

* Average of 3 determinations by ELISA technique.

**Table 6:** Interactions and binding scores of screened phytochemical compounds within EGFR TK Domain binding pocket (3W33).

Compound	Binding Scores (kcal/mol)	Hydrogen Bond Interactions	Distance (Å)	Hydrophobic Interactions	Distance (Å)
Retinol	−9.30			VAL 726	3.83, 3.84
				LYS 745	3.76
				ALA 743	3.75, 3.94
				LEU 718	3.58
				LEU 844	3.87
Podocarp-7-en-3β-ol, 13β-methyl-13-vinyl	−8.20			VAL 726	3.81
				ALA 743	3.88
				LYS 745	3.78
				THR 790	3.43
				THR 854	3.81
Megastigmatrienone	−7.00	LEU 777	−2.54	ALA 743	3.84
				LYS 745	3.77
				LEU 858	3.91
Cedrol	−7.00			LEU 718	3.78
				VAL 726	3.83
				MET 793	3.82
				CYS 797	3.91
				LEU 844	3.12
β-Sitosterol	−10.60	CYS 797	2.95	LEU 718	3.21
				PHE 856	3.02
				VAL 726	3.91
				ALA 743	3.25
				THR 790	3.12
				LEU 844	3.78
				THR 790	3.81
Docosanol	−6.20	ASP 800	3.41	THR 790	3.81
				LEU 718	3.43
				ARG 841	3.65
				LEU 844	3.82
				LYS 745	3.48
				ALA 743	3.18
W19 [49]	−10.50	MET 793 SER 720	2.51 2.29	PHE 856	3.88
				LEU 788	3.82
				LYS 745	3.48
				LEU 844	3.93
				ALA 743	3.81
				VAL 726	3.68

## Data Availability

The data used during the current study will be available from the corresponding author on reasonable request.

## Abbreviations

The following abbreviations are used in this manuscript:

A549	Human lung cancer cell line
ACC	Activity of complement-mediated cytotoxicity
ADCC	Cell-mediated antibody-dependent cytotoxicity
ATCC	American type culture collection
CC50	Half-maximal cytotoxic concentration
CPE	Virus-induced cytopathic effect
DAPI	4′,6-diamidino-2-phenylindole
DMEM	Dulbecco’s modified Eagle medium
DMSO	Dimethyl sulfoxide
EGFR-TK	Epidermal Growth Factor Receptor Tyrosine Kinase Domain
GC-MS	Gas chromatographic–mass spectrometry
H1N1	Influenza A virus
HSV-2	Herpes Simplex virus
HCT116	Human colorectal cancer cell line
HepG2	Human hepatocellular cancer cell line
IC_50_	Half-maximal inhibitory concentration
MCF7	Human breast cancer cell line
MDB	Microsoft Access Database
MDCK	Madin–Darby canine kidney cells
MOE	Molecular Operating Environment
MTT	3-(4,5-dimethylthiazol-2-yl)-2,5-diphenyl-2H-tetrazolium bromide
M. Wt.	Molecular weight
NIST	National Institute of Standards and Technology
NK	Natural killer
PBS	Phosphate-buffered saline
RMSD	Root mean square deviation
RPMI	Roswell Park Memorial Institute Medium
RT	Room temperature
SD	Standard deviation
W19	4-{[4-(1-benzothiophen-4-yloxy)-3-chlorophenyl]amino}-N-(2-hydroxyethyl)-8,9-dihydro-7H-pyrimido[4,5-b]azepine-6-carboxamide
